# Pseudo Chediak-Higashi Anomaly

**DOI:** 10.4274/Tjh.2011.0010

**Published:** 2013-06-05

**Authors:** Zekai Avcı, Barış Malbora, Namık Özbek

**Affiliations:** 1 Başkent University Faculty of Medicine, Department of Pediatric, Hematology, Ankara, Turkey; 2 Dr. Sami Ulus Maternity and Children’s Hospital, Department of Pediatric Hematology, Ankara, Turkey; 3 Başkent University Faculty of Medicine, Department of Pediatric Hematology and Oncology, Ankara, Turkey

**Keywords:** Pseudo Chediak-Higashi anomaly, Acute myeloblastic leukemia, child

A 9-year-old girl was admitted to hospital with a 1-month history of fever, weight loss, epistaxis, and abdominal pain. The girl’s parents were non-consanguineous. Her medical history was unremarkable. Upon admission she weighed 27 kg (25th-50th percentile), was 131 cm tall (50th percentile), and was pale. Physical examination showed multiple cervical and inguinal microlymphadenopathies, without hepatosplenomegaly. Complete blood count findings were as follows: hemoglobin 7.9 g/dL; white blood cell count: 12.3x109/L; platelet count: 9.2×109/L. Her peripheral blood smear showed 43% blast cells, 42% lymphocytes, 8% monocytes, 6% neutrophils, and 1% eosinophils. Bone marrow aspiration showed hypercellularity, with 45% myeloblasts, 15% promyelocytes, 7% myelocytes, 4% metamyelocytes, 1% eosinophils, 2% neutrophils, 15% lymphocytes, and 11% normoblasts. Wright staining of a bone marrow smear showed large (2-4 μm) round-to-oval, hyaline-structured eosinophilic granules (pseudo Chediak-Higashi [PCH] granules) within 15% of the blast cells, promyelocytes, and myelocytes (Figure 1). Some of the granules appeared to be in vacuoles, giving them a haloed appearance. The blasts were myeloperoxidase (MPO)-positive, and periodic acid-Schiff (PAS)-negative. Immunophenotypic analysis of the leukemic cells showed proliferation of CD13 (75%), CD33 (59%), CD34 (95%), CD117 (68%), and HLA DR (44%). 

The patient was diagnosed with as type M2 acute myeloid leukemia (AML). Cytogenetic analysis of a bone marrow sample showed 45,XX,t(8;21)(q22;q22). Cerebrospinal fluid biochemistry was normal, without detectable cells, based on cytocentrifuge analysis. The patient was treated according to the Berlin-Frankfurt-Munster (BFM) 2004 treatment protocol for AML. She is being followed without chemotherapy for two years and she does not have any problems.

PCH anomaly was first described in 1964 by Didisheim et al. [1]. Later, VanSlyck and Rebuck [2] reported similar granules in the leukemic cells of 2 patients with AML-M4, and used the term, pseudo Chediak-Higashi anomaly of acute leukemia, because of the resemblance of the granules to those seen in patients with inherited Chediak-Higashi syndrome. PCH anomaly is characterized by the presence of large cytoplasmic eosinophilic granules in leukemic blast cells, promyelocytes, and myelocytes. It is most often observed in patients with AML subtypes M2, M3, M4, and M5, but it is also associated with chronic myeloid leukemia, myelodysplastic syndrome, and mixed-lineage leukemias [1,2,3,4,5,6,7]. Most reported cases of PCH anomaly in leukemia patients are in adults; there are only a few reported childhood cases. 

Ultrastructural studies of PCH anomaly have shown that the granules are strongly MPO positive, with variable positivity for PAS, Sudan black, and high-iron diamine, and without an obviously crystalline structure [5,8]. Electron microscopic studies suggest that these granules are formed by the fusion of azurophilic granules [9]. Some studies propose that these granules should be considered a morphological variant of Auer bodies [10]; however, the pathophysiology of PCH anomaly remains unknown. The clinical significance of this abnormality has yet to be been determined, because PCH anomaly is not always associated with disseminated intravascular coagulation or any other characteristic clinical picture. Additional research is required to establish the therapeutic and prognostic relevance of PCH anomaly.

## Figures and Tables

**Figure 1:**
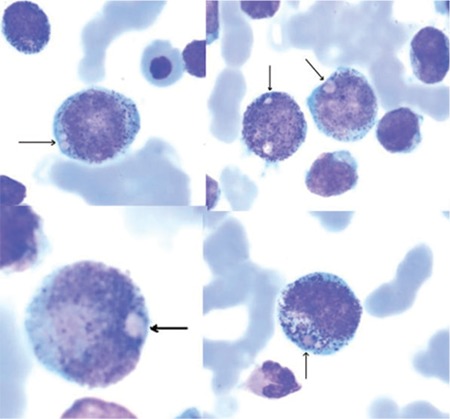
Wright staining of a bone marrow smear shows large eosinophilic granules (PCH granules) within the blast and myeloid cells.

## References

[1] Didisheim P, Trombold JS, Vandervoort LE, Mibashan RS (1964). Acute promyelocytic leukemia with fibrinogen and factor V deficiencies. Blood.

[2] Van Slyck EJ, Rebuck JW (1974). Pseudo-Chediak-Higashi anomaly in acute leukemia. A significant morphologic corollary.. Am J Clin Pathol.

[3] Powari M, Varma N, Varma S, Komal HS (2000). Pseudo-Chediak Higashi anomaly in an Indian patient with acute myeloid leukemia (AML-M2). Am J Hematol.

[4] Symes PH, Williams ME, Flessa HC, Srivastava AK, Swerdlow SH (1993). Acute promyelocytic leukemia with the pseudo-Chediak-Higashi anomaly and molecular documentation of t(15;17) chromosomal translocation. Am J Clin Pathol.

[5] Rao S, Kar R, Saxena R (2009). Pseudo Chediak-Higashi anomaly in acute myelomonocytic leukemia. Indian J Pathol Microbiol.

[6] Tsai IM, Tsai CC, Ladd DJ (1977). Pseudo-Chediak-higashi anomaly in chronic myelogenous leukemia with myelofibrosis. Am J Clin Pathol.

[7] Gallardo R, Kranwinkel RN (1985). Pseudo-Chédiak-Higashi anomaly. Am J Clin Pathol.

[8] Ahluwalia J, Kumar V, Trehan A, Marwaha RK, Garewal G (2004). The psuedo-Chediak-Higashi anomaly: an unusual staining pattern in an Indian child with acute myeloid leukemia. Pediatr Hematol Oncol.

[9] Tulliez M, Vernant JP, Breton-Gorius J, Imbert M, Sultan C (1979). Pseudo-Chediak-Higashi anomaly in a case of acute myeloid leukemia: electron microscopic studies. Blood.

[10] Payne CM, Harrow EJ (1983). A cytochemical and ultrastructural study of acute myelomonocytic leukemia exhibiting the pseudo-Chediak-Higashi anomaly of leukemia and “splinter-type” Auer rods. Am J Clin Pathol.

